# Dr. Charles M. Brasted

**Published:** 1909-03

**Authors:** 


Dr. Charles M. Brasted, of Hornell, N. Y., who graduated at the University of Buffalo, 1881, died suddenly January 1, 1909, while making an emergency professional visit in his home city, aged 50 years.

